# BRCA1 Induces Major Energetic Metabolism Reprogramming in Breast Cancer Cells

**DOI:** 10.1371/journal.pone.0102438

**Published:** 2014-07-10

**Authors:** Maud Privat, Nina Radosevic-Robin, Corinne Aubel, Anne Cayre, Frédérique Penault-Llorca, Geoffroy Marceau, Vincent Sapin, Yves-Jean Bignon, Daniel Morvan

**Affiliations:** 1 Jean Perrin Comprehensive Cancer Center and ERTICA EA4677 Research Team, University of Auvergne, Clermont-Ferrand, France; 2 INRA, UMR 1019, UNH, Clermont-Ferrand, France; 3 Laboratoire de biochimie médicale, Centre de biologie, CHU de Clermont-Ferrand, Clermont-Ferrand, France; The Norwegian University of Science and Technology (NTNU), Norway

## Abstract

The hypermetabolic nature of cancer cells and their increased reliance on “aerobic glycolysis”, as originally described by Otto Warburg and colleagues, are considered metabolic hallmarks of cancer cells. BRCA1 is a major tumor suppressor in breast cancer and it was implicated in numerous pathways resulting in anticarcinogenic functions. The objective of our study was to address specific contributions of BRCA1 to the metabolic features of cancer cells, including the so-called “Warburg effect”. To get a comprehensive approach of the role of BRCA1 in tumor cell metabolism, we performed a global transcriptional and metabolite profiling in a BRCA1-mutated breast cancer cell line transfected or not by wild-type BRCA1. This study revealed that BRCA1 induced numerous modifications of metabolism, including strong inhibition of glycolysis while TCA cycle and oxidative phosphorylation tended to be activated. Regulation of AKT by BRCA1 in both our cell model and BRCA1-mutated breast tumors was suggested to participate in the effect of BRCA1 on glycolysis. We could also show that BRCA1 induced a decrease of ketone bodies and free fatty acids, maybe consumed to supply Acetyl-CoA for TCA cycle. Finally increased activity of antioxidation pathways was observed in BRCA1-transfected cells, that could be a consequence of ROS production by activated oxidative phosphorylation. Our study suggests a new function for BRCA1 in cell metabolic regulation, globally resulting in reversion of the Warburg effect. This could represent a new mechanism by which BRCA1 may exert tumor suppressor function.

## Introduction

Breast cancer susceptibility gene 1 (*BRCA1*) is a major breast cancer suppressor gene and the most frequently mutated gene in hereditary breast cancer. In addition, BRCA1 down-regulation was shown in a large part of sporadic breast cancer [1], [2]. The BRCA1 protein has multiple but incompletely understood molecular and cellular functions. It plays a critical role in the cellular response to stress and functions as a sensor of DNA damage. BRCA1 is implicated in DNA double-strand break repair, cell cycle control, transcriptional regulation, ubiquitination, apoptosis and resistance to anticancer agents [3].

BRCA1 has been shown to interact with many key cellular proteins, including p53, c-Myc, AKT [4], HIF1α [5], [6], and acetyl-CoA carboxylase (ACCA) [7]. BRCA1 may exert its effects by controlling the stability and degradation of target proteins, including pACCA, HIF1α, and pAKT [4], [5], [7].

The conversion of glucose to lactate, which can occur in hypoxic normal cells, persists in cancer tissues despite the presence of oxygen that would normally inhibit glycolysis. This aerobic glycolysis, also named the Warburg effect, represents a major metabolic hallmark of cancer cells. It is now known that sustained aerobic glycolysis in cancer cells is linked to activation of oncogenes or loss of tumor suppressors. An increasing body of evidence establishes the role of HIF1α, Akt, Myc, and p53 in the regulation of glycolysis [8], [9], [10]. Recently, new tumor suppressor genes encoding proteins involved in metabolic pathways have been discovered, notably those involved in citrate metabolism [11], including *sdha/c* (succinate dehydrogenase) in paragangliomas [12], [13], [14], [15], and *idh1/2* (isocitrate dehydrogenase) in low and high grade gliomas [16], [17]. These findings on the regulation of tumor metabolism by oncogenes or tumor suppressors have renewed the interest for metabolism as a field of discovery for biomarkers and therapeutic targets [18], [19].

However, the impact of BRCA1 on tumor cell metabolism remains unclear. BRCA1 has been shown to regulate *de novo* fatty acid synthesis [7], and protect tumor cells against oxidative stress [20], [21]. To uncover the role of BRCA1 on tumor metabolism, combined transcriptional and metabolic profiling was performed in breast cancer cells expressing or not BRCA1. The combination of the transcriptome and the metabolome has been recently exploited with success [22], [23]. In this article, metabolism-targeted transcriptomics and untargeted metabolomics were analyzed in combination to characterize major traits of BRCA1-induced metabolic reprogramming.

We found that wild-type BRCA1 transfection in mutant cells induced numerous modifications of metabolism, including strong inhibition of glycolysis, while TCA cycle and oxidative phosphorylation tended to be activated. Increased activity of antioxidation pathways and alteration of fatty acid and inositol metabolism were also induced by BRCA1. Our study thus provided evidence of implication of BRCA1 in the regulation of bioenergetic metabolism, another mechanism by which BRCA1 may exert its tumor suppressor function.

## Materials and Methods

### Biological materials

The SUM1315 human breast cancer cell line was obtained from Asterand (Royston, Hertfordshire, UK) and was grown in Ham’s F12 medium according to the supplier’s instructions at 37°C in a humidified atmosphere containing 5% CO_2_. SUM1315-BRCA1 and SUM1315-LXSN cell lines were obtained by stable transfection of SUM1315 human breast cancer cells with an empty LXSN plasmid, or a *BRCA1*-encoding LXSN plasmid as previously described [24]. These cell lines were grown in a selective culture medium with 721.5 µM G418 (Sigma-Aldrich, St. Louis, MO).

Tumor samples from 29 patients with invasive breast carcinoma were collected from the Centre Jean Perrin tumorbank. The collection of biopsies was performed in compliance with the Helsinki Declaration and all patients signed a form of no objection to the use of their tissue samples for research.

### Proliferation and apoptosis assays

Proliferation assays were performed using the Sulforhodamine B technique. Briefly, 1 to 10 days after cell seeding, 10% trichloroacetic acid (Sigma-Aldrich, St. Louis, MO) was added to the culture medium in order to fix cells for one hour at 4°C. Cells were then stained with 0.4% SRB (Sigma-Aldrich, St. Louis, MO) in 1% acetic acid and washed with 1% acetic acid. SRB was then solubilised in Tris base (10 mM; pH 10.5) and 540 nm optical density was determined using a microplate reader.

Percentage of apoptotic cells was determined using AnnexinV-PE apoptosis detection kit (BD Pharmingen). Briefly, cells were washed in phosphate buffered saline, harvested by trypsinisation and incubated for 15 min with AnnexinV-Phycoerythrin and 7 Amino-actinomycin D as recommended by the manufacturer. Analysis of at least 10,000 events was performed using a Coulter Epics XL flow cytometer (Beckman Coulter).

### RNA extraction and quantitative RT-PCR

Eight biological replicates were extracted from cell cultures using RNA PLUS reagent (QBiogene) according to the manufacturer’s protocol. Quality of RNAs was checked using the 2100 BioAnalyzer (Agilent Technologies). Five microgram RNA was then reverse-transcribed using First-strand cDNA synthesis kit (GE Healthcare).

A total of 93 genes (Table S1) were selected from a bibliographical review on tumor metabolism and the KEGG pathway database (www.genome.jp/kegg/). The aim was to choose genes coding for enzymes implicated in the major metabolic pathways as classified by the KEGG database. We have preferentially selected genes that had previously deserved attention in cancer metabolism literature. TaqMan low density arrays (Applied Biosystems) were used to evaluate mRNA expression using an ABI Prism 7700 thermal cycler (Applied Biosystems).

Relative gene expression was determined for each gene using the comparative threshold cycle method. Briefly, mean threshold cycles (Ct) of all replicates were calculated and normalized to internal control genes (geometric average of Actin and 18S) Ct to give dCT. Expression in SUM1315-BRCA1 cells relative to SUM1315-LXSN cells was determined by the ddCT method (ddCT = SUM1315-BRCA1 [dCT] − SUM1315-LXSN [dCT]). Fold changes were determined as 2(−ddCT).

### Aqueous and organic dual cell extraction

At least 4 biological replicates of extraction was done in an ice-cold environment. Cells were suspended in 2 mL of methanol/chloroform (2∶1, v/v) and ultrasonicated for 1 min. 500 µL of purified water/chloroform (1∶1, v/v) were added and the organic and aqueous phases were separated by centrifugation (1500 g, 20 min, 4°C). Both phases were evaporated under argon flux, then lyophilized and stored at –80°C.

### Dual ^1^H-NMR spectroscopy and liquid chromatography-mass spectrometry (LC-MS)

Parallel analyses of water soluble extracts by ^1^H-NMR spectroscopy and liquid chromatography-mass spectrometry (LC-MS) were performed using the Metabolic Profiler dual analyzer (Bruker Daltonik GmbH, Bremen, Germany).

Samples were prepared using a Gilson 215 liquid handler (Gilson Medical Electronics, Villiers le Bel, France). The dried extracts were rehydrated in a solution containing 0.15 M phosphate buffer (pH 7.2), 5 mM sodium trimethylsilyl[2,2,3,3-^2^H_4_]propionate (TSP), and 2 mM NaN_3_ in D_2_O.

1D ^1^H-NMR spectra were obtained using the NOESYGPPR1D pulse sequence (Bruker), which is a presaturation experiment for water suppression incorporating the first increment of the NOESY pulse sequence and the z-axis spoiler gradient sequence. The pulse sequence was of the form *d1*-*Π*/2-*t1*-*Π*/2-*tm*-*Π*/2, where *Π*/2 represented a 90° hard pulse, *d1* the relaxation delay (4 s), *t1* a short delay (6.5 µs), and *tm* the mixing time (10 ms). The 90° hard pulse was automatically calibrated for each sample before analysis to avoid peak phase and baseline distortions of spectra. The resonance of H_2_O (4.78 ppm) was selectively irradiated with a continuous wave low power pulse during *d1* and *tm*. For each sample, 512 transients were collected into 64 k data points over a spectral width of 20 ppm at 27°C. Prior to Fourier transformation, the free-induction decay was smoothed using an exponential function resulting in a line-broadening of 0.3 Hz. A minimal baseline correction was applied to spectra using the Topspin Version 2.0 software (Bruker Biospin, Karlsruhe, Germany). Spectra were referenced to the TSP resonance (0.00 ppm). Then spectra were normalized to the integral of signal between 1.0 and 4.5 ppm, and identified signals were integrated. Signals were assigned to metabolites from literature tables and publicly available databases of chemical shifts, j-coupling constants and peak structures.

LCMS spectra were obtained using a microTOF ESI-TOF mass spectrometer (Bruker Daltonik GmbH, Bremen, Germany) coupled to a HPLC system (Agilent 1200 Series High Performance Autosampler, Waldbronn, Germany) controlled by the HyStar chromatography software (Bruker Daltonik GmbH). A mobile phase system consisting of water and acetonitrile (each containing 0.1% formic acid) was used, and separation was performed with an RP-18 column (Zorbax Eclipse XDB-C18 4.6×150 mm, 3.5 µ particle size, Agilent). Data sets were acquired in positive electrospray ionization mode in a scan range from 50 to 800 *m/z* at a sampling rate of 1 Hz. Sodium formate solution was used for calibration and injected at the beginning of each chromatographic run. Quality control samples and blank runs were interspersed between the samples. MS raw data were converted into NetCDF files using the Metabolic Profiler software suite (Bruker Daltonik GmbH). Subsequent data processing was performed using XCMS (http://metlin.scripps.edu) including retention time alignment, matched filtration, peak detection, and peak matching. Then peaks were integrated. Metabolites were identified from publicly available databases and a home-made LCMS library of standards.

In addition, ^1^H-NMR spectroscopy was performed in intact cells according to the technique in reference [25]. Briefly, NMR spectroscopy was performed on a small-bore 500-MHz Bruker Avance DRX spectrometer equipped with a high resolution magic angle spinning (HRMAS) probe. Intact cell pellets (5–10×10^6^ cells) were set into 4 mm-diameter, 50-µL, free-volume, zirconium oxide rotor tubes. Rotors were spun at 4 kHz and cooled at 4°C, using the BCU-05 temperature unit. One-dimensional ^1^H-NMR spectra were obtained using a nuclear Overhauser enhancement spectroscopy sequence with low-power water-signal presaturation (NOESYPR) during both the 3.8-sec relaxation delay and the 100-ms mixing time of the sequence. The spectral width was 12 ppm with 16,384 complex data points and 32 transients. This resulted in 2 min 50 sec acquisition duration. After Fourier transformation, spectra were phased.

### High performance thin layer chromatography (HPTLC)

40 µL of cellular lipid extract in chloroform was used for each sample. Lipids were separated by thin-layer chromatography on a silica gel plate (Merck, Darmstadt, Germany). HPTLC plates were used after prewashing with a mixture of chloroform-methanol (1∶1, v/v) followed by heating at 110°C for 10 min. Lipid extracts were applied under a flow of nitrogen on the HPTLC plate using a Linomat IV apparatus (CAMAG, Muttenz, Switzerland) and separated using the following sequential development system: development to half final distance in methyl acetate–chloroform–propanol–methanol–0.25% KCl in water (25∶25∶25∶10∶9, v/v) followed by full development in hexane-diethylether-acetic acid (80∶20∶2, v/v) to resolve non-polar lipids. Quantification was performed after staining (10% CuSO4 w/v in 8% H_3_PO_4_ (v/v)) and charring at 160°C. The plates were scanned and quantification was obtained using density measurements with SIGMA scan pro 5.0 software.

### Gas-chromatography flame spectrometry (GCFS)

Fatty acid methyl esters were prepared via basic transesterification of sodium methoxide in methanol (Sigma-Aldrich, St. Louis, MO) followed by acid transesterification of boron trifluoride in methanol 14%. Analyses were performed using a TRACE gas chromatograph (Thermo Fisher Scientific, Waltham, MA) equipped with a flame ionization detector. Helium was used as carrier gas at a constant pressure of 264 kPa, and a split/splitless injector (1 µL) was used in splitless mode. The fatty acid methyl esters were analyzed using a CP-Sil 88 capillary column (100 m/0.25 mm internal diameter/0.20 µm film thickness) (Varian, Palo Alto, CA). The oven temperature program ran from 70°C to 225°C (4 steps separated by 3 ramps). The temperature of the injector was 250°C; that of the detector was 280°C.

### Gas measurement

To establish hypoxic conditions, cells were plated in 60 mm dishes and placed in a hypoxic chamber (O_2_sensBox, Adelbio, Clermont-Ferrand, France) flushed with a 1%O_2_/5%CO_2_/94%N2 gas mixture for 4H and 20 H at 37°C. Control cells were incubated in normoxic atmosphere containing 5% CO_2_ for 20H at 37°C. One mL of medium was removed using a syringe and a capillary tube which was closed immediately after in order to stay in anaerobic conditions. pO_2_ was then immediately measured in medium with a Synthesis 15 Blood Gas Analyzer (Instrumentation Laboratory, Le Pré St Gervais, France).

### Biochemical measurement

One million of cells were seeded in T75 flask and grown during 72H. Media were then collected and centrifuged at 1950 g for 5 min at 4°C. Concentrations of lactate and glucose were determined on an automated clinical chemistry analyzer (Dimension Vista 1500, Siemens Healthcare Diagnostics) based on enzymatic colorimetric assays as recommended by the provider. Metabolite concentration in culture medium was normalized to the metabolite concentration measured in medium without cells.

### Western blotting

Proteins were extracted from cells with a lysis buffer containing 50 mM Tris (pH 7.5), 5 mM EDTA, 150 mM NaCl, 0.25% Triton X-100 and 1 mM DTT. Protease inhibitors (Complete protease inhibitor cocktail, Roche) and phosphatase inhibitors (50 mM NaF, 1 mM Na3VO4) were added to the basic buffer. Twenty five microgram proteins were electrophoresed on a SDS–polyacrylamide gel and transferred onto a nitrocellulose membrane. After a one-hour blocking in Tris buffered saline Tween 0.1% (TBST) containing 5% milk, membranes were incubated overnight at 4°C with the appropriate dilution of antibody. Membranes were then washed three times in TBST and incubated for one hour with horseradish peroxidase-conjugated secondary antibody (1∶2000). Detection was then performed with the ECL detection system (GE Healthcare). Mouse OP92 (Ab-1) anti-BRCA1 primary antibody was purchased from Oncogene Research (San Diego, CA). C14 (sc-6521) anti- HK2 and N20 (sc-49996) IDH1 primary antibodies were purchased from Santa Cruz Biotechnology (Santa Cruz, CA, USA), and p-AKT(ser473, ID9271), AKT (ID9272) from Cell Signaling Technologies (Danvers, MA, USA). Anti-FASN antibody (ID610962) came from BD Biosciences (Le Pont de Claix, France) and anti-HIF1α (H1alpha67) from Novus Biologicals (Littleton, CO, USA).

### Tumor and cell immunohistochemistry

Tumors were fixed in buffered formalin and embedded in paraffin. Cells were fixed in Preservcyt solution (Thinprep) and cytoblocks were prepared with Shandon Cytoblock kit (Thermo Scientific). HIF1α, total AKT and pAKT status were determined by immunohistochemistry on 3 µm-thick sections. The antibodies came from Abcam (Cambridge, UK) for HIF1α (ab463), and from Ozyme (St Quentin Fallavier, France) for total AKT (C67E7) and pAKT (Ser473). For HIF1α, total AKT and pAKT immunostaining, the antibodies were diluted 1/750, 1/100 and 1/50 and were incubated 32 min at room temperature, 1 h at 37°C and 2 h at room temperature respectively. Immunostainings were performed in a BenchmarkXT fully automatized stainer (Ventana Medical Systems, Tucson, USA). The immunostainings were scored semi-quantitatively by an expert pathologist under a light upright microscope. Scores ranged from 0 to 3 and positivity was considered for scores of 2 and 3.

### Statistical handling of data

Gene expression intensity and metabolite levels were normalized to the average of the SUM1315-LXSN cell line (set to 1, which preserved the control group variability). Fold variations were calculated for the SUM1315-BRCA1 cell line. Univariate analyses between groups were performed using the bilateral Mann-Whitney test, unless specified.

Hierarchical ascending classification was performed on the whole set of data using the GeneCluster and Treeview 1.6 software (University of California). The similarity criterion was the Spearman coefficient correlation and clustering was based on average linkage.

## Results

**Figure 1:**
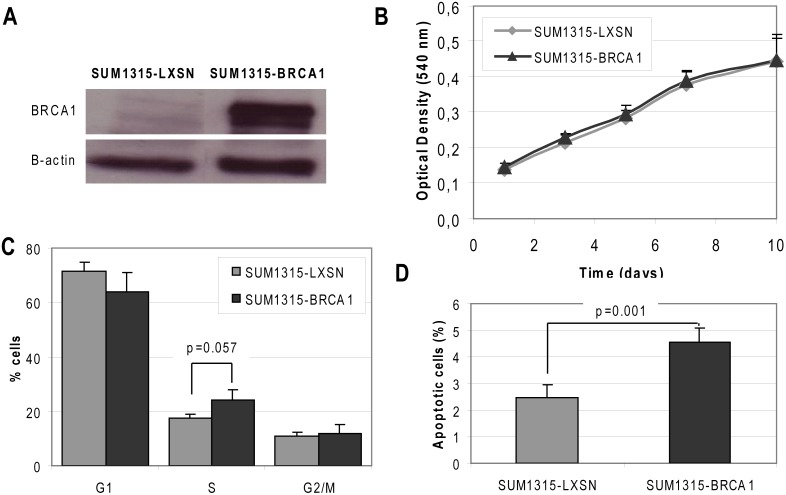
Phenotypic characterization of cell lines. A. Expression of BRCA1 was analysed by Western blotting using anti-BRCA1 antibody. Mouse anti-BRCA1 primary antibody was purchased from Oncogene Research (San Diego, CA). Actin was used as a loading control. B. Proliferation experiments were performed using the Sulforhodamine B technique. Data are means of three independent experiments + SEM. C. Cycle distribution was determined using flow cytometry. Data are means of three independent experiments + SEM. D. Percentage of apoptotic cells was determined at 48 h using the Annexin V test. Data are presented as means of 3 independent replicates + SEM. *: p<0.05.

The SUM1315 cell line was chosen for our study since it carries the c.68_69delAG mutation of the *BRCA1* gene [26]. In the Breast Cancer Information Core database (http://research.nhgri.nih.gov/bic/), this mutation is the most common mutation found in *BRCA1* mutant breast cancer families. SUM1315 cells were stably transfected with a LXSN plasmid encoding wild-type BRCA1. We previously showed that unlike cells transfected with the empty plasmid (SUM1315-LXSN cells), *BRCA1*-transfected cells (SUM1315-BRCA1 cells) expressed the 220 kDa nuclear BRCA1 protein ([24] and Figure 1A). In order to characterize SUM1315-LXSN and SUM1315-BRCA1 cell line phenotypes, we analyzed proliferation, apoptosis and cell cycle distribution. Although BRCA1 transfection resulted in a slightly increased apoptosis population (Figure 1D, p = 0.001), this apoptotic rate stay rather low and the two cell lines have the same proliferation rate (Figure 1B) and cell cycle distribution (Figure 1C).

### Global metabolic phenotype of cell lines

**Figure 2:**
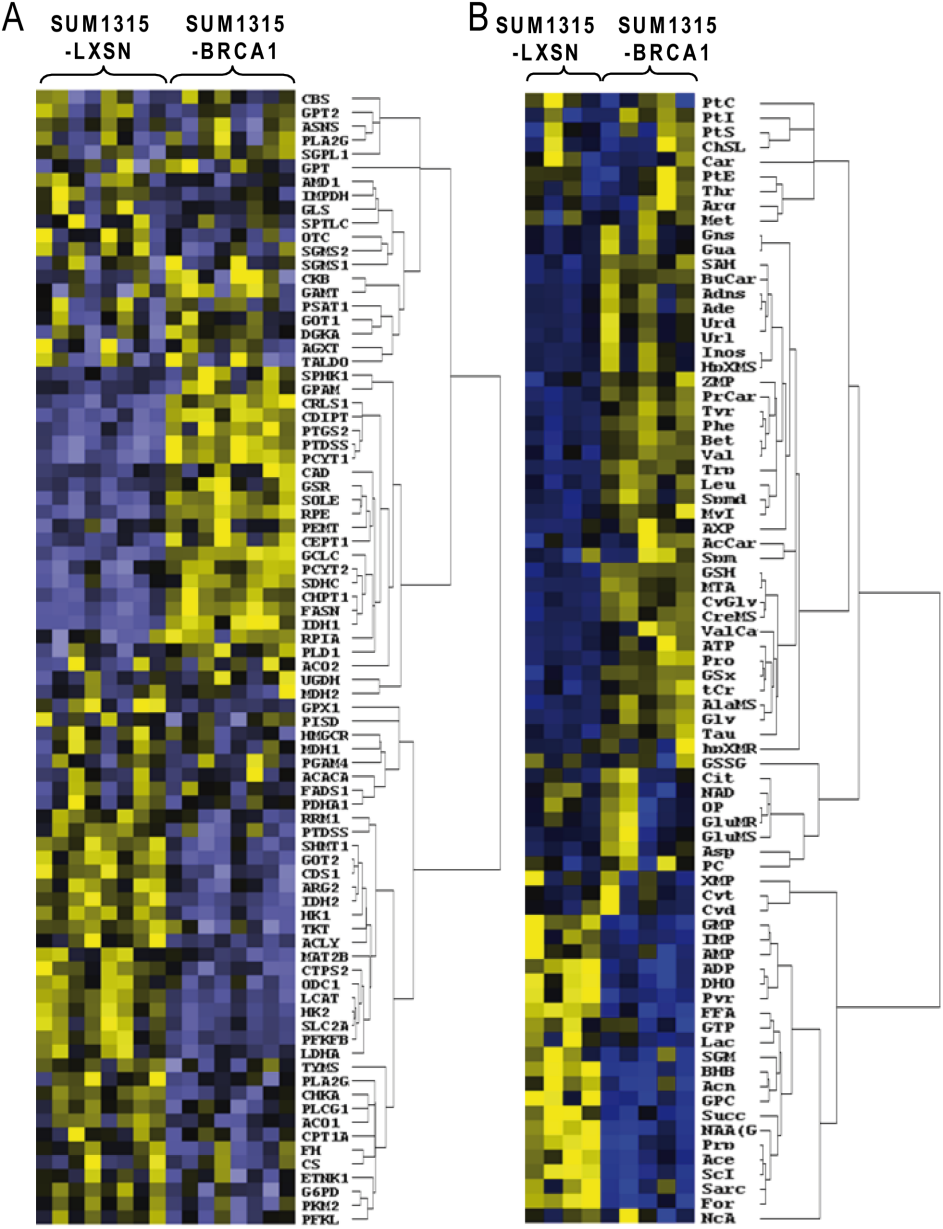
Regulation of metabolism by BRCA1 protein in SUM1315-LXSN and SUM1315-BRCA1 cell model. A. Gene expression study: mRNA of SUM1315-LXSN and SUM1315-BRCA1 cells were extracted and analyzed on TaqMan Low Density Arrays as described in materials and methods. The replicates represent independent culture experiments. Low levels of expression are represented in blue and high levels of expression in yellow. B. The major metabolites observed by ^1^H-NMR and by LC-MS were analyzed by hierarchical clustering**.** The replicates represent independent culture experiments. Small quantities of metabolites are represented in blue and high quantities in yellow.

To elucidate the role of BRCA1 on tumor metabolism, combined transcriptional and metabolic profiling was performed in SUM1315-LXSN and SUM1315-BRCA1 breast cancer cells. Hierarchical ascending classification revealed transcripts and metabolites that co-varied between samples (Figure 2). The two cell lines were clearly discriminated in two different clusters, suggesting an important impact of BRCA1 on metabolism.

The expression of selected genes (a total of 93 genes) coding major metabolic enzymes and pathways was obtained by quantitative RT-PCR (Figure 2A and table S1). The most strongly down-regulated cluster of transcripts in SUM1315-BRCA1 included those involved in glycolysis (SLC2A1, HK1, HK2, PFKFB3, p<0.001; LDHA, p<0.01), TCA cycle (IDH2, GOT2, p<0.001), nitrogen metabolism (ODC1, ARG2, p<0.001) and lipid metabolism (LCAT, CDS1, p<0.001). The most strongly up-regulated cluster included transcripts involved in antioxidant response (IDH1, GCLC, p<0.001; GSR), lipid metabolism (FASN, PCYT1A, PTDSS1, p<0.001), and TCA cycle (SDHC, p<0.05).

Metabolites were obtained using ^1^H-NMR spectroscopy and LCMS, and completed with GCFS and HPTLC data for lipid metabolites (Figure 2B and Tables S2, S3, S4, and S5). The metabolite cluster with the lowest levels in SUM1315-BRCA1 contained ketone bodies (acetone, p<0.01; N-acétylaspartate, acetate, β-hydroxybutyrate, propionate, p<0.001), bioenergetic intermediates (pyruvate, p<0.01; succinate, p<0.05), lipids (free fatty acids, p<0.01), nucleic acid derivatives (ADP, p<0.01) and an unexpected sugar, scylloinositol (p<0.001). The metabolite cluster with the highest levels in SUM1315-BRCA1 cells included glutathione cycle derivatives (GSH, cysteinglycine, total glutathione, p<0.001), nucleic acids (ATP, p<0.01), and another stereoisomer of inositol, myoinositol (p<0.001).

The effects of BRCA1 on metabolism in the SUM1315 cell line included:

- down-regulated glycolysis and increased TCA cycle, coupled to oxphos, at least at SDHC level;
- disturbed lipid metabolism with a decrease of free fatty acids;
- up-regulated glutathione metabolism;

Further studies were then undertaken to explore these metabolic changes.

### BRCA1 increased TCA cycle and oxidative phosphorylation activity and decreased activity of glycolysis

**Figure 3:**
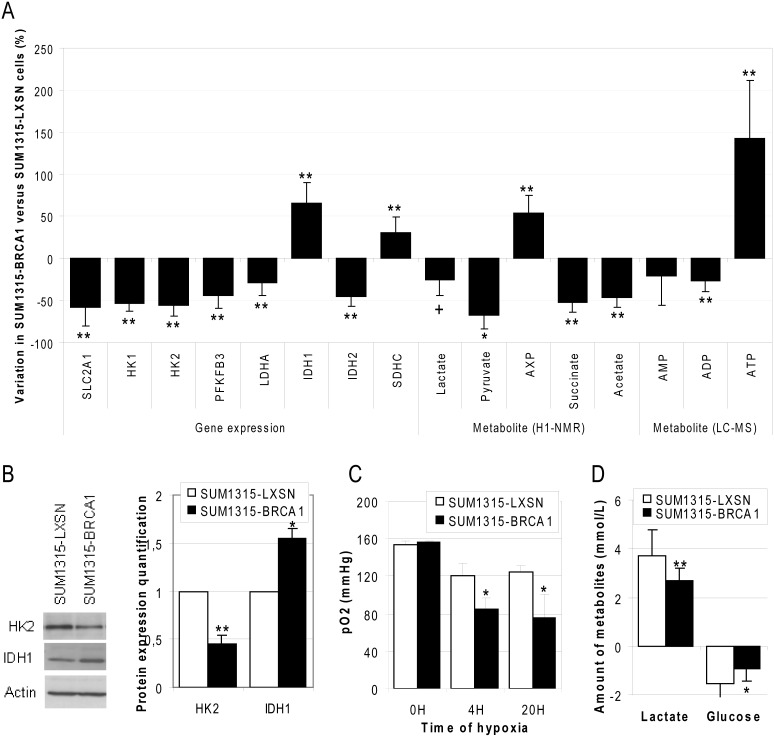
Role of BRCA1 in energetic metabolism. A. The major enzymes and metabolites involved in energy metabolism were quantified by q-RT-PCR, ^1^H-NMR and by LC-MS. Data are presented as means of at least 3 independent replicates + SEM. *: p<0.05; **: p<0.01, +: p = 0.06 for lactate, SUM1315-BRCA1 vs SUM1315-LXSN cell lines. B. Analyses of HK2 and IDH1 enzymes was performed by Western Blot and quantified by ImageJ software. Data are presented as means of 3 independent replicates + SEM. *: p<0.05; ***: p<0.001, SUM1315-BRCA1 vs SUM1315-LXSN cell lines. C. The oxygen partial pressure was measured in the culture medium of SUM1315-LXSN and SUM1315-BRCA1 cell lines subjected to hypoxic atmosphere for the indicated time. *: p<0.05, SUM1315-BRCA1 vs SUM1315-LXSN cell lines. D. Production of lactate and consumption of glucose were measured in cell culture media by enzymatic colorimetric assays. Data are presented as means of 3 independent replicates + SEM.

Five genes highly implicated in the glycolysis pathway were repressed (*SLC2A1*, *HK1*, *HK2, PFKFB3* and *LDHA*, Figure 3A). The *SLC2A1* gene codes for the glucose transporter 1 (Glut1), which allows glucose entry in the cells; hexokinases, coded by *HK1* and *HK2* genes, catalyze the first reaction of glycolysis; the PFKFB3 enzyme is a major regulator of glycolysis as it increases the steady-state concentration of a potent PFK-1 allosteric activator, fructose-2,6-bisphosphate (Fru-2,6-BP); *LDHA* gene codes for a lactate dehydrogenase, which catalyses the interconversion of pyruvate, the final step of glycolysis, and lactate.

Pyruvate and lactate, the final glycolysis products, were both decreased by BRCA1 transfection (Figure 3A, p = 0.061 for lactate and p = 0.014 for pyruvate), while ATP levels in SUM1315-BRCA1 tumor cells were increased. These changes were accompanied by increase in the transcription of the enzyme SDHC, which converts succinate into fumarate and a consistent decreased levels of succinate. The *SDHC* gene has been shown to be a major tumor suppressor gene in mitochondria [27]. It encodes a key enzyme in the TCA cycle and oxidative phosphorylation of mitochondria that allows transformation of succinate into fumarate.

Then, two enzymes involved in the conversion of isocitrate in oxoglutarate, IDH1 in the cytosol and IDH2 in the mitochondria were inversely regulated. In SUM1315-BRCA1, up-regulation of IDH1 could supply NADPH for glutathione reduction, the major cell defense against oxidative stress. IDH1 increase may also participate in a metabolic loop to replenish mitochondria with oxoglutarate. IDH2 is a reverse enzyme which role could be to convert oxoglutarate to isocitrate [28]. Down-regulation of IDH2 in SUM1315-BRCA1 cells could then allow activation of TCA cycle.

In order to confirm regulation of mRNA at protein levels, HK2 and IDH1 protein levels were quantified by Western Blot (Figure 3B). As shown on the quantification graph, these protein levels revealed significant regulation of BRCA1 (around 50% decreased expression of HK2 and 50% increased expression of IDH1). These results thus confirmed the regulation observed at mRNA level by Q-RT-PCR.

The energetic pathway used by cells appears to be modified by BRCA1 transfection. We thus analyzed the breast cancer cell respiration by measuring pO_2_ in cell culture media (Figure 3C). O_2_ is indeed consumed only by mitochondrial respiratory chain which is coupled to TCA cycle and oxidative phosphorylation. In aerobic conditions, O_2_ is in excess and we could not see any difference between the two cell lines. In reduced O_2_ conditions, SUM1315-BRCA1 cells consumed more O_2_ than SUM1315-LXSN cells. This result confirmed that BRCA1 restoration induced increase of TCA cycle and OXPHOS, which consume O_2_.

To check BRCA1 involvement in glycolysis regulation, we performed enzymatic glucose and lactate measures in SUM1315-LXSN and SUM1315-BRCA1 culture media. We already showed that the BRCA1 transfection did not affect cell proliferation (Figure 1B). We could thus measure how much glucose was consumed and how much lactate was produced by the 2 cell lines (Figure 3D). As expected, BRCA1-mutated cells consumed more glucose (p = 0.01) and released more lactate (p = 0.005) in cell culture medium than BRCA1 wild-type cells.

Some major glycolysis regulating pathways have already been shown to interact with BRCA1 pathway. BRCA1-mutated tumors have been shown to more frequently overexpress HIF1α than sporadic tumors [6]. We thus tested HIF1α expression in our cell model. HIF1α protein was stabilized more efficiently in BRCA1 mutated cells but only when the cells were treated by the hypoxia mimetic CoCl_2_ (figure S1). Moreover, no significant differences were observed in HIF1α expression when BRCA1 mutated and BRCA1 wild-type tumors were compared (data not shown).

The oncogene AKT can also stimulate the transcription of a number of genes that encode the proteins that mediate the glycolysis pathway [29]. In our cell model, we found a higher level of phosphorylated form of AKT in SUM1315-LXSN than in SUM1315-BRCA1 cells (Figure S2A). As already suggested in other studies [4], [30], BRCA1 mutation was also correlated with high level of phosphorylated form of AKT in several other cell lines and tumour models (Figure S2C and S2D). Finally, AKT inhibition by B2311 [31] reduces lactate production in SUM1315-LXSN cells (Figure S2B). In SUM1315-BRCA1 cells, AKT inhibition by B2311 was less efficient and lactate production was not modified. This supports the notion that wild-type BRCA1 contributes to control PI3K signalling, thereby reducing glycolytic flux.

### BRCA1 increased activity of antioxidation pathways

**Figure 4:**
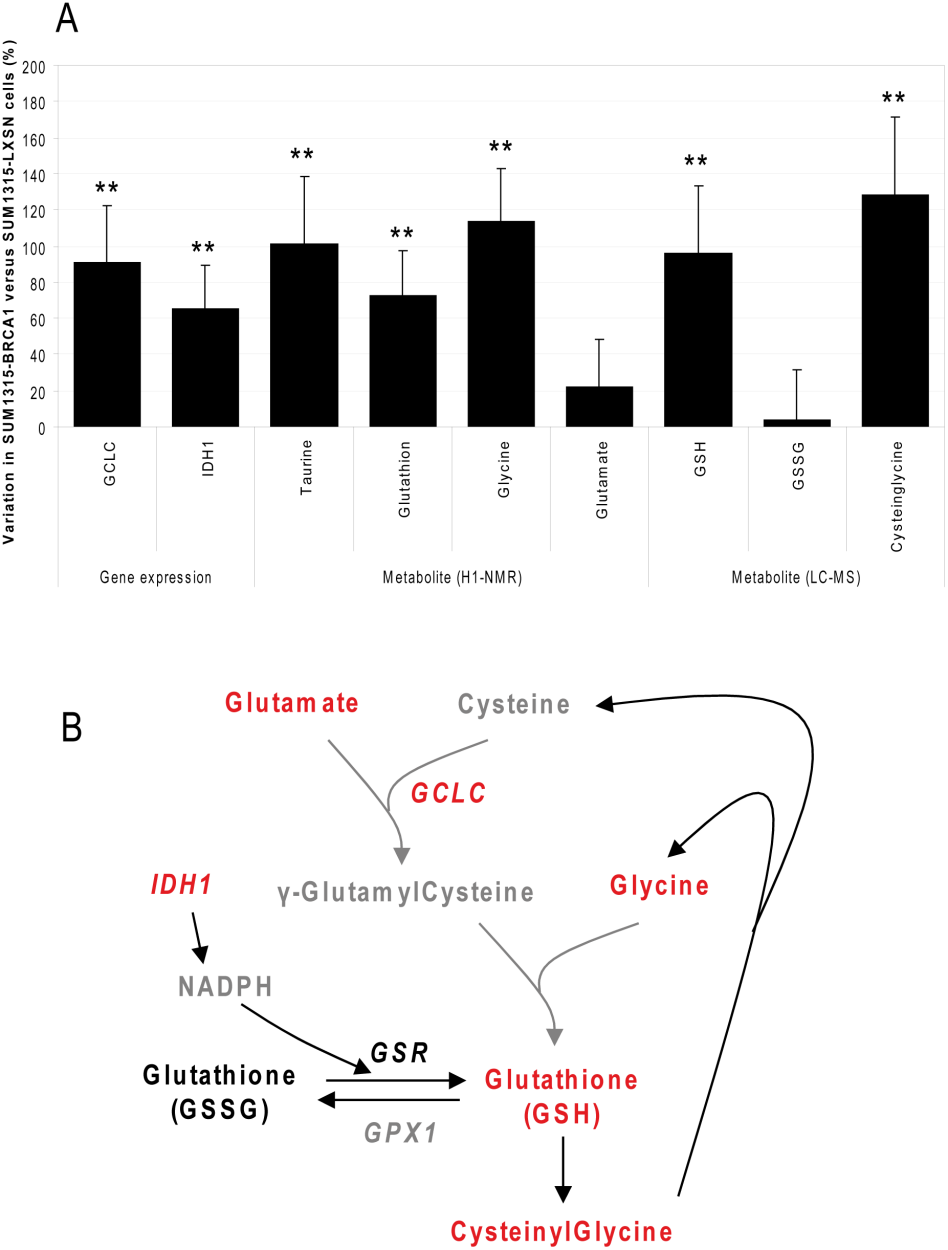
BRCA1-induced increased activity of antioxidation pathways. A. The major enzymes and metabolites involved in antioxidation metabolism were quantified by q-RT-PCR, ^1^H-NMR and by LC-MS. Data are presented as means of at least 3 independent replicates + SEM. *: p<0.05; **: p<0.01, SUM1315-BRCA1 vs SUM1315-LXSN cell lines. B. Schematic representation of glutathione metabolism and its regulation by BRCA1. Genes (*italicized*) in red: overexpression. Metabolites: grey, not detected; red, increased.

In glutathione metabolism, *GCLC* (glutamate cysteine ligase, or gamma-glutamylcysteine synthetase), the first rate-limiting enzyme of glutathione synthesis, was increased in SUM1315-BRCA1 cells (Figure 4A). Detoxifying metabolites taurine and glutathione, together with the amino acids forming glutathione (glutamate and glycine), were increased in SUM1315-BRCA1. IDH1 increase could also contribute to reduction of glutathione by providing NADPH. LC-MS analyses showed that only the reduced form of glutathione (GSH) was increased following BRCA1 transfection. Reduced glutathione is the major endogenous antioxidant produced by cells, participating directly in the neutralization of free radicals and reactive oxygen compounds. BRCA1 may then influence cell redox status (Figure 4B).

### BRCA1 modifies the metabolism of lipids

**Figure 5:**
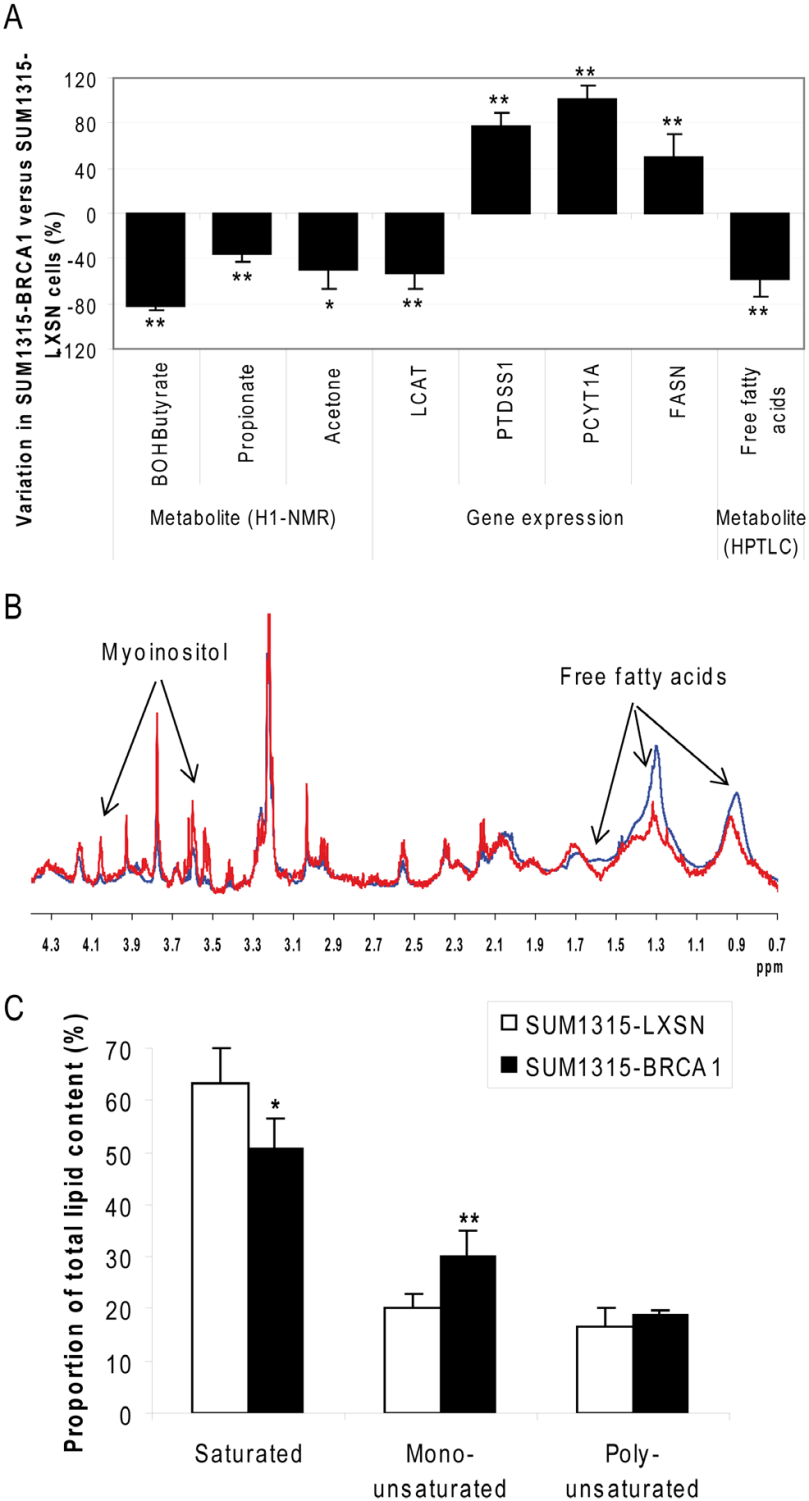
Reprogramming of lipid metabolism induced by BRCA1. A. The major enzymes and metabolites involved in lipid metabolism were quantified by ^1^H-NMR, quantitative RT-PCR and HPTLC. Data are presented as means of at least 3 independent replicates + SEM. *: p<0.05; **: p<0.01, SUM1315-BRCA1 vs SUM1315-LXSN cell lines. BOHButyrate: β-hydroxybutyrate. B. Intact whole cell pellets were analyzed by ^1^H-NMR spectroscopy in order to reveal differential metabolites the most concentrated in breast tumor cells. Spectra were normalized according to a published procedure [25]. Representative spectra of intact SUM1315-LXSN (blue) and SUM1315-BRCA1 (red) cells are shown. Arrows: most concentrated differential signals. C. Fatty acid composition was analyzed by GCFS. Results of major groups are given as percentage of total lipids. Data are presented as means of at least 3 independent replicates + SEM. *: p<0.05; **: p<0.02, SUM1315-BRCA1 vs SUM1315-LXSN cell lines.

First, the ^1^H-NMR study only concerned the soluble fraction extracted from cells so hydrophobic lipids could not be observed. Nevertheless, β-hydroxybutyrate, propionate and acetone, all related to the biosynthesis of ketone bodies, were downregulated by BRCA1 (Figure 5A). Ketone bodies are water-soluble compounds that are produced as by-products when fatty acids are broken down for energy.

Lipid metabolism transcripts were modified by BRCA1 transfection, some being down-regulated (LCAT) and others up-regulated (PTDSS1, PCYT1A and FASN) (Figure 5A). LCAT, PTDSS1 and PCYT1A are enzymes implicated in the metabolism of phospholipids but HPTLC analyses of organic cellular extracts showed no differences in this lipid quantification in SUM1315-LXSN and SUM1315-BRCA1 cells (table S4). FASN, one of the key lipogenic enzymes, was slightly up-regulated by BRCA1 at transcriptionnal level. This is in contradiction with our HPTLC data which showed a dramatic decrease of free fatty acids in SUM1315-BRCA1 cells (Figure 5A: –58% compared to SUM1315-LXSN, p<0.01). In order to understand this contradiction, we studied protein regulation of FASN and ACCA, another important enzyme of free fatty acid metabolism. ACCA and FASN proteins were slightly up-regulated by BRCA1, although over several experiments, these findings were not significant (figure S3).

Pellets of intact SUM1315-LXSN and SUM1315-BRCA1 cells were also analyzed by HRMAS ^1^H-NMR spectroscopy. With intact cells, the highest discriminating signals were those at 1.30 and 1.64 ppm, corresponding to fatty acids, which were decreased by approximately 78% in SUM1315-BRCA1 cells (Figure 5B).

Finally, lipid composition was obtained by GCFS analyses (table S5). A decreased proportion of saturated fatty acids was showed in SUM1315-BRCA1, together with an increased proportion of monounsaturated fatty acids (Figure 5C).

## Discussion

Metabolism-related transcriptomics and metabolomics were combined to explore BRCA1-induced metabolic reprogramming of breast cancer cells.

A major finding was downregulation of glycolysis in BRCA1-expressing tumor cells. All glycolysis indicators were decreased by about 50% in *BRCA1* wild-type cells when compared to *BRCA1* mutant cells. Five major enzymes of this pathway, including HK2 and PFKFB3, and both pyruvate and lactate were down-regulated by BRCA1 transfection. The HK2 gene is up-regulated in cancer cells and is sometimes considered as the major gene responsible for Warburg’s glycolysis increase. Several glycolytic inhibitors that target HK2 have been proposed in cancer treatment [32], [33], [34]. PFKFB3 also plays a key role in glycolysis regulation by elevating the concentration of Fru-2,6-BP, the most potent glycolysis stimulator. As this metabolic conversion has been suggested to be a hallmark of cancer, PFKFB3 has emerged as a novel target for cancer chemotherapy [34], [35]. Moreover, we found that analyses of glucose consumption and lactate production confirmed a decrease in glycolytic activity in BRCA1-transfected cells. The entire pathway was thus modified in breast cancer cells expressing BRCA1 wild-type protein.

It is likely that BRCA1 exerts its effects on glycolysis by several pathways and one of them could be the AKT pathway. It has already been shown that AKT could be inactivated by BRCA1 [4], [36]. This major oncoprotein can induce glycolysis via multiple mechanisms, including expression and activation of hexokinases (AKT can phosphorylate HK2) and activation of PFK enzymes [29]. Our data show that BRCA1 mutation seems to be correlated with high AKT phosphorylation levels. This suggests that a mutation in BRCA1 could remove AKT inhibition and thus cause glycolysis to be up-regulated, eventually enabling tumor transformation.

Despite the decrease in glycolysis, BRCA1 induced a global increase in cellular ATP levels. This ATP can only come from the only other source of energy in the cell: the TCA cycle and oxidative phosphorylation. Moreover, the TCA cycle seemed specifically regulated: conversion of succinate to fumarate was promoted by SDHC up-regulation in SUM1315-BRCA1 cells. This reaction is essential to the electron transport chain, which results in ATP production. Succinate, which accumulates in BRCA1-mutated cells as a result of low SDH level, can exit the mitochondria and activate HIF1α [37], [38]. This protein is a transcription factor which regulates transcription of nearly all the genes involved in glycolysis [39], [40]. Furthermore BRCA1 mutant tumours more frequently overexpress HIF1α than sporadic breast tumours [6]. We also observed a better stabilisation of HIF1α by cobalt, a hypoxia mimetic, in BRCA1-mutated cells. Overexpression of SDHC and concomitant succinate decrease in BRCA1-transfected cells could thus explain both down-regulation of glycolysis (via degradation of HIF1α) and increased of cellular ATP (via activation of TCA cycle and oxphos).

Acetyl-CoA is an essential metabolite of the TCA cycle, but BRCA1-induced glycolysis decrease resulted in less acetyl-coA production. To allow TCA activation, this loss must then be compensated by other pathways. Ketone bodies and free fatty acids decrease could be markers of this increased need for acetyl-coA. Ketone bodies, including acetone and β-hydroxybutyrate, can be reconverted to acetyl-CoA to produce energy via the citric acid cycle. β-oxidation of fatty acids is another way to produce acetyl-coA. BRCA1-transfected cells could have increased fatty acid and ketone body catabolism in order to produce Acetyl-CoA and activate the TCA cycle coupled to oxidative phosphorylation.

It has already been shown that BRCA1 is able to decrease free fatty acids [7], [41], [42]. Moreau et al. showed that BRCA1 protein stabilizes pACCA, the phosphorylated and inactive form of ACCA, and this finding was proposed to be the origin of reduced *de novo* fatty acid synthesis. This could be a mechanism by which BRCA1 leads to inhibition of free fatty acid production in order to preserve acetyl-coA to supply the TCA cycle.

In addition, BRCA1-mutated breast tumor cells are more sensitive to oxidative stress than BRCA1-expressing cells, as discussed below This mechanism was correlated to the production of lipid droplets [43], which could further explain the increased total fatty acid content in BRCA1-mutated cells.

It has indeed been reported that BRCA1 induces the expression of glutathione-S-transferase, and other antioxidant genes, and confers resistance to oxidative stress [20]. This study confirms upregulation of antioxidation pathways in BRCA1-expressing cells, including NADPH-producing systems such as IDH1, and glutathione biosynthesis (increased mRNA levels of GCLS, increased GSH levels, and decreased GSSG-to-GSH ratio). Classically, the activation of oxidative phosphorylation leads to production of reactive oxygen species (ROS), a mechanism participating in the upregulation of antioxidation pathways. The increased activity of the anti-oxidant system could then arise from the BRCA1-induced reversion of the Warburg phenotype.

High levels of glutathione, increased activity of antioxidation pathways, and maintenance of cells in a reduced status may induce protection against DNA, protein and membrane lipid damage in BRCA1-expressing cells [20], [44].

**Figure 6:**
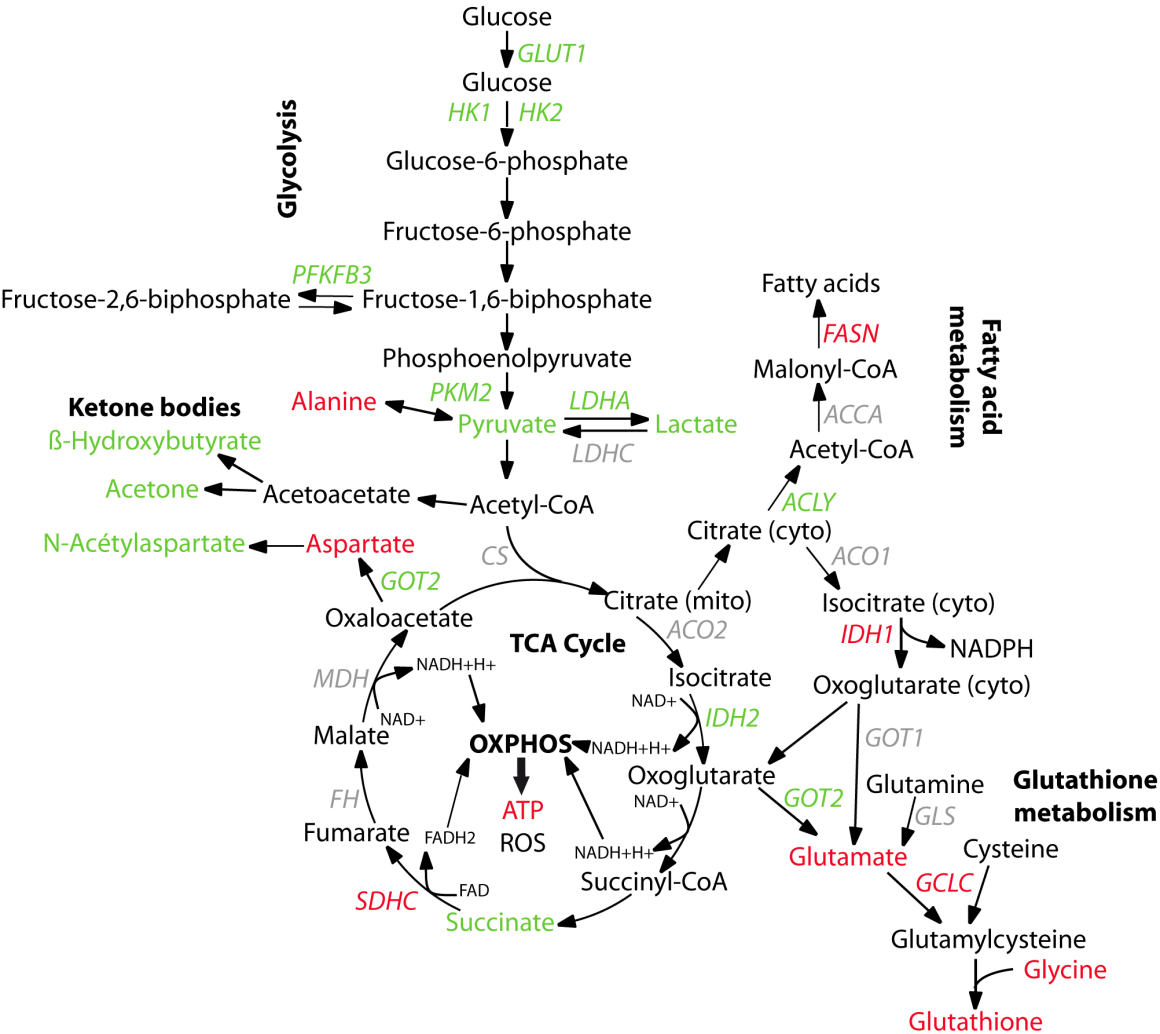
Schematic representation of global metabolism regulation by wild-type BRCA1 transfection. Enzymes or metabolites up-regulated in SUM1315-BRCA1 cells compared to SUM1315-LXSN cells are represented in red whereas down-regulated ones are presented in green.

Warburg hypothesized in 1956 that increased glycolysis combined with suppression of mitochondrial function is the most fundamental metabolic change in malignant transformation [45]. Our results suggest that BRCA1 could reverse this “Warburg effect”. Through several adaptative pathways, wild-type BRCA1 indeed induced down-regulation of glycolysis, together with increased mitochondrial energy producing pathways (Figure 6). This new global function in metabolism could represent another mechanism by which BRCA1 exerts its tumor suppressive function.

## Supporting Information

Figure S1 **Quantification of HIF1α was performed by Western blot in SUM1315-LXSN and SUM1315-BRCA1 cells treated or not by the hypoxia mimetic CoCl_2_.** Anti-HIF1α antibody came from Novus Biologicals (Littleton, CO).
(TIF)

Figure S2 **Implication of AKT in BRCA1 role in energetic metabolism.** A. The pathway implicated in energetic metabolism modification was tested by Western blotting of AKT. Quantification was performed using ImageJ software. Data are presented as means of 3 independent replicates + SEM. B. The effectiveness of the AKT inhibition by 0.5 µM of AKT inhibitor IV (B2311, Sigma) was tested by Western blot. It was evaluated by ImageJ software at 67% of inhibition in SUM1315-LXSN cell line and 47% of inhibition in SUM1315-BRCA1 cell line (data not shown). Production of lactate and consumption of glucose were measured in cell culture media. Data were normalized to untreated control and are presented as means of at least 3 independent replicates + SEM. ***: p<0.001, SUM1315-BRCA1 vs SUM1315-LXSN cell lines. C. Immunocytochemistry studies were performed to characterize BRCA1 wild-type cell lines (MCF7 and MDA-MB-231) and BRCA1 mutated cell lines (HCC1937 and SUM149). Pictures show representative p-AKT stainings in these cell lines. D. AKT phosphorylation was analysed in 17 BRCA1 wild type tumors (a) and in 12 BRCA1 mutated tumors (b) by immunohistochemistry. The staining was considered positive when at least 10% of the cells showed a membranous signal. The table summarises the results and pictures show representative p-AKT stainings in tumor examples. A p<0.0001 was calculated by the Khi^2^ statistical test.
(TIF)

Figure S3 **Quantification of FASN, phosphorylated and total forms of ACACA enzymes was performed by Western Blot and ImageJ software.** Anti-p-ACACA(Ser79) and ACACA antibodies came from Cell Signaling Technologies (Danvers, MA) and anti-FASN from BD Biosciences (Le Pont de Claix, France). Data are presented as means of 3 independent replicates + SEM.
(TIF)

Table S1 **Taqman RTQ-PCR arrays data.** mRNA of SUM1315-LXSN and SUM1315-BRCA1 cells were extracted and analysed on TaqMan Low Density Arrays as described in procedures. Data were calculated as intensities with the 2^(−ΔΔCT)^ method and are presented as means of 8 independent replicates + SEM. *: p<0.05; **: p<0.02; ***: p<0.01, SUM1315-BRCA1 vs SUM1315-LXSN cell lines.
(TIF)

Table S2 ^1^H-**NMR spectroscopy data were obtained as described in experimental procedures.** Data were normalised to 1 for SUM1315-LXSN condition and are presented as means of at least 4 independent replicates + SEM. *: p<0.05; **: p<0.02; ***: p<0.01, SUM1315-BRCA1 vs SUM1315-LXSN cell lines.
(TIF)

Table S3 **LC-MS data were obtained as described in experimental procedures.** Data were normalised to 1 for SUM1315-LXSN condition and are presented as means of at least 4 independent replicates + SEM. *: p<0.05; **: p<0.02; ***: p<0.01, SUM1315-BRCA1 vs SUM1315-LXSN cell lines.
(TIF)

Table S4 **HPTLC data were obtained as described in experimental procedures.** Data were normalised to 1 for SUM1315-LXSN condition and are presented as means of at least 3 independent replicates + SEM. *: p<0.05; **: p<0.02; ***: p<0.01, SUM1315-BRCA1 vs SUM1315-LXSN cell lines.
(TIF)

Table S5 **GCFS data were obtained as described in experimental procedures.** Data are presented as a proportion of total lipid content and as means of 3 independent replicates + SEM. *: p<0.05; **: p<0.02; ***: p<0.01, SUM1315-BRCA1 vs SUM1315-LXSN cell lines.
(TIF)
