# Supplementary figures and images for: BRCA1 Induces Major Energetic Metabolism Reprogramming in Breast Cancer Cells
Source: PLoS One. 2014 Jul 10;9(7):e102438. doi: 10.1371/journal.pone.0102438 (PMC4092140; doi:10.1371/journal.pone.0102438)

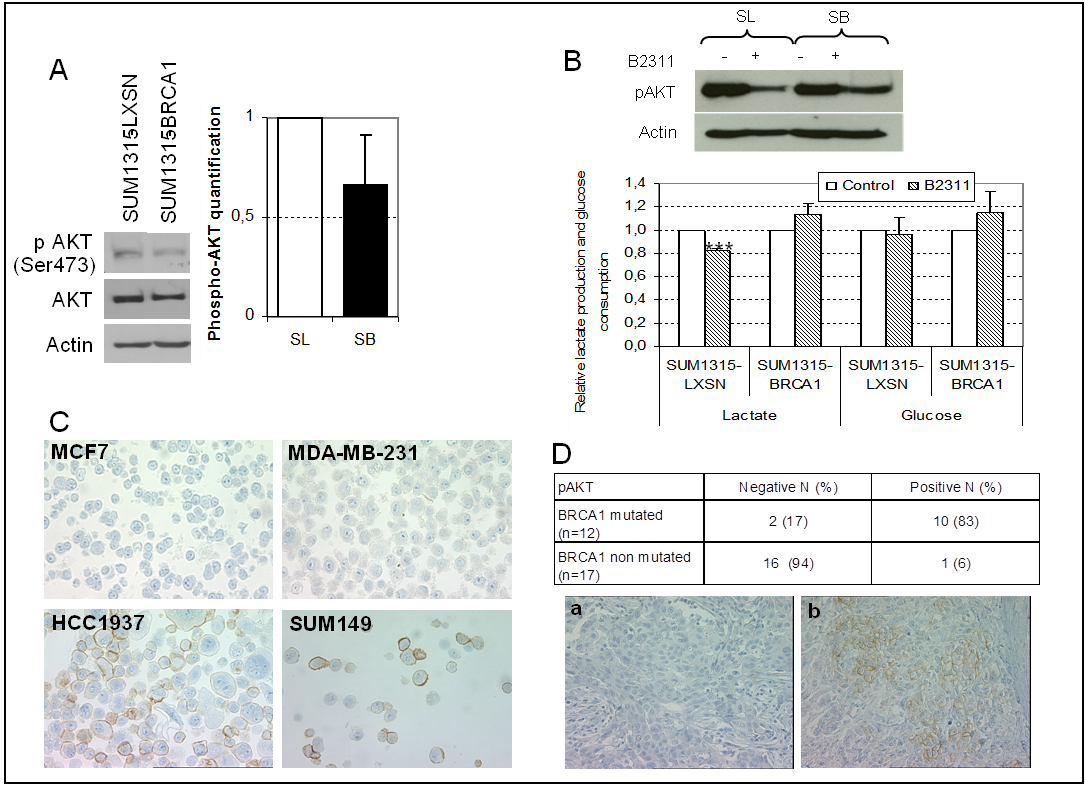

Supplement: Figure S2 — Implication of AKT in BRCA1 role in energetic metabolism. A. The pathway implicated in energetic metabolism modification was tested by Western blotting of AKT. Quantification was performed using ImageJ software. Data are presented as means of 3 independent replicates + SEM. B. The effectiveness of the AKT inhibition by 0.5 µM of AKT inhibitor IV (B2311, Sigma) was tested by Western blot. It was evaluated by ImageJ software at 67% of inhibition in SUM1315-LXSN cell line and 47% of inhibition in SUM1315-BRCA1 cell line (data not shown). Production of lactate and consumption of glucose were measured in cell culture media. Data were normalized to untreated control and are presented as means of at least 3 independent replicates + SEM. ***: p<0.001, SUM1315-BRCA1 vs SUM1315-LXSN cell lines. C. Immunocytochemistry studies were performed to characterize BRCA1 wild-type cell lines (MCF7 and MDA-MB-231) and BRCA1 mutated cell lines (HCC1937 and SUM149). Pictures show representative p-AKT stainings in these cell lines. D. AKT phosphorylation was analysed in 17 BRCA1 wild type tumors (a) and in 12 BRCA1 mutated tumors (b) by immunohistochemistry. The staining was considered positive when at least 10% of the cells showed a membranous signal. The table summarises the results and pictures show representative p-AKT stainings in tumor examples. A p<0.0001 was calculated by the Khi2 statistical test. (TIF) [file pone.0102438.s002.tif]

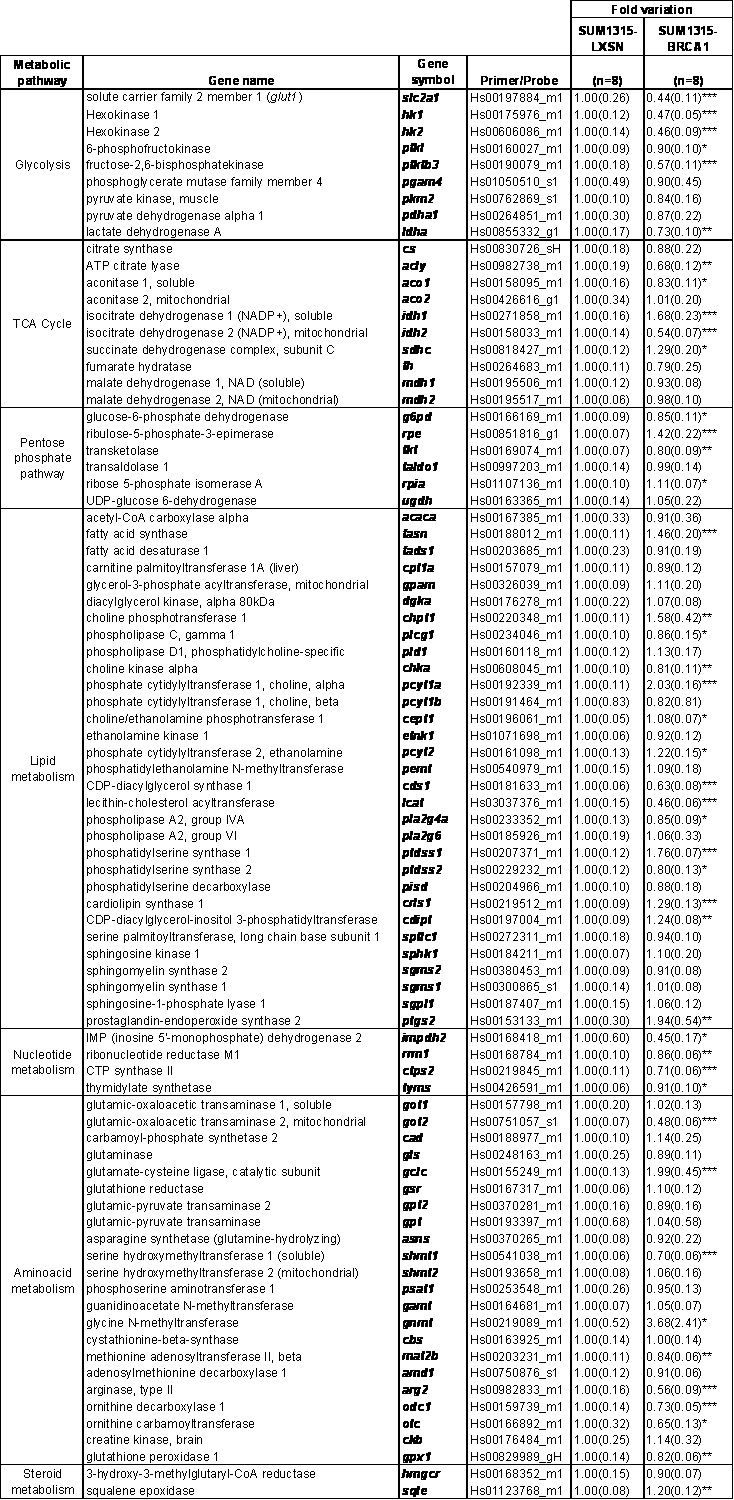

Supplement: Table S1 — Taqman RTQ-PCR arrays data. mRNA of SUM1315-LXSN and SUM1315-BRCA1 cells were extracted and analysed on TaqMan Low Density Arrays as described in procedures. Data were calculated as intensities with the 2(−ΔΔCT) method and are presented as means of 8 independent replicates + SEM. *: p<0.05; **: p<0.02; ***: p<0.01, SUM1315-BRCA1 vs SUM1315-LXSN cell lines. (TIF) [file pone.0102438.s004.tif]

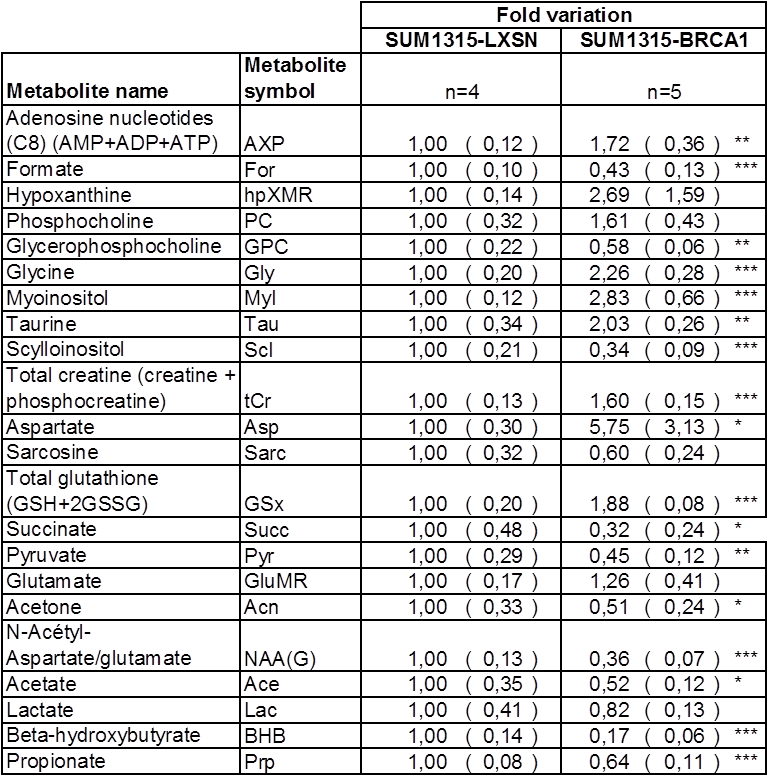

Supplement: Table S2 — 1H-NMR spectroscopy data were obtained as described in experimental procedures. Data were normalised to 1 for SUM1315-LXSN condition and are presented as means of at least 4 independent replicates + SEM. *: p<0.05; **: p<0.02; ***: p<0.01, SUM1315-BRCA1 vs SUM1315-LXSN cell lines. (TIF) [file pone.0102438.s005.tif]

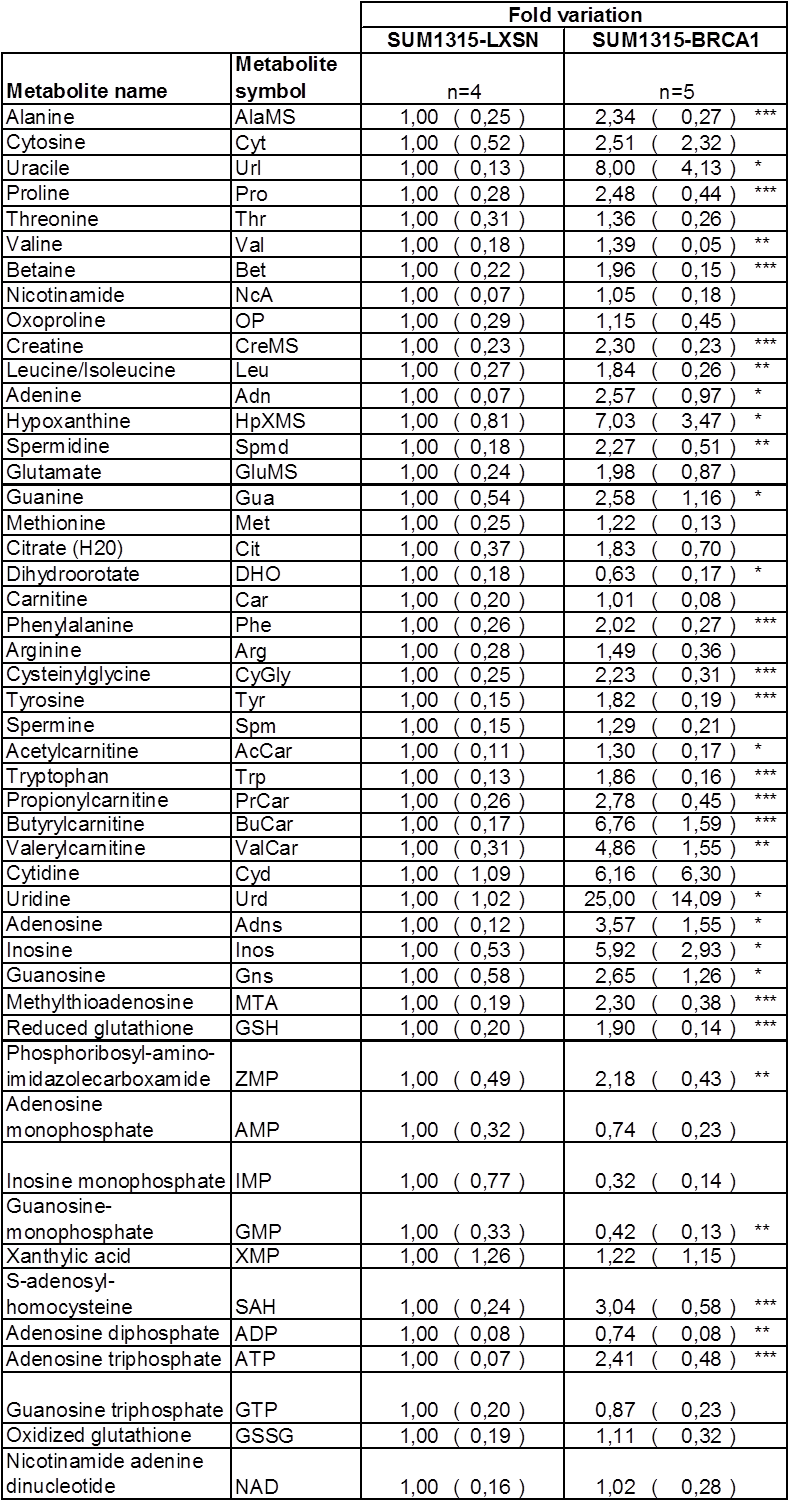

Supplement: Table S3 — LC-MS data were obtained as described in experimental procedures. Data were normalised to 1 for SUM1315-LXSN condition and are presented as means of at least 4 independent replicates + SEM. *: p<0.05; **: p<0.02; ***: p<0.01, SUM1315-BRCA1 vs SUM1315-LXSN cell lines. (TIF) [file pone.0102438.s006.tif]

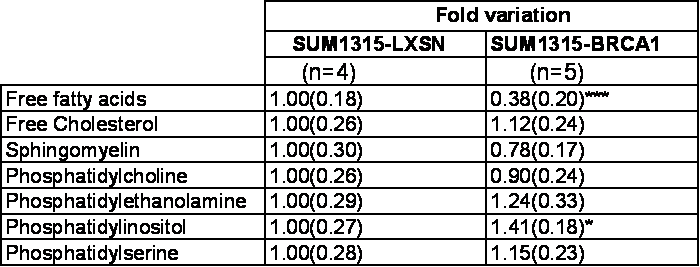

Supplement: Table S4 — HPTLC data were obtained as described in experimental procedures. Data were normalised to 1 for SUM1315-LXSN condition and are presented as means of at least 3 independent replicates + SEM. *: p<0.05; **: p<0.02; ***: p<0.01, SUM1315-BRCA1 vs SUM1315-LXSN cell lines. (TIF) [file pone.0102438.s007.tif]

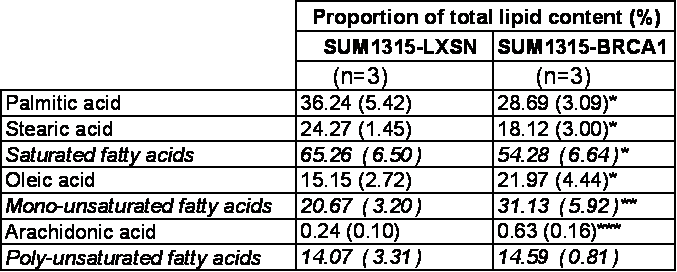

Supplement: Table S5 — GCFS data were obtained as described in experimental procedures. Data are presented as a proportion of total lipid content and as means of 3 independent replicates + SEM. *: p<0.05; **: p<0.02; ***: p<0.01, SUM1315-BRCA1 vs SUM1315-LXSN cell lines. (TIF) [file pone.0102438.s008.tif]
